# Melanopsin-Mediated Pupillary Responses in Bipolar Disorder: A Non-Systematic Review

**DOI:** 10.1192/j.eurpsy.2025.1074

**Published:** 2025-08-26

**Authors:** M. O. Pires, H. J. Gomes, R. M. Freitas, C. Freitas, M. Albuquerque

**Affiliations:** 1 Department of Psychiatry and Mental Health, Guarda Local Health Unit, Guarda; 2 Department of Psychiatry and Mental Health, Nordeste Local Health Unit, Bragança; 3 Integrated Responsability Center - Mental Health, Hospital Pedro Hispano, Matosinhos, Portugal

## Abstract

**Introduction:**

Melanopsin-expressing intrinsically photosensitive retinal ganglion cells (ipRGCs) are critical regulators of circadian rhythms and various non-image-forming visual functions, such as the pupillary light reflex (PLR), which adjusts the amount of light entering the eye. ipRGCs, through their direct responsiveness to circadian light, influence both circadian entrainment and neurophysiological processes that affect mood regulation. Emerging evidence suggests that melanopsin-mediated PLR may be disrupted in mood disorders, including bipolar disorder (BD) and even patients with increased bipolar disorder-traits. Given the well-documented association between circadian dysregulation and BD pathophysiology, it is hypothesized that changes in ipRGC function may contribute to the neurobiological mechanisms underlying BD.

**Objectives:**

The primary aim of this review is to synthesize and evaluate the existing body of literature on melanopsin-mediated pupillary responses in individuals with bipolar disorder. Specifically, this review seeks to assess the extent to which these responses are altered in BD and to explore the potential utility of melanopsin-driven PLR as a biomarker for mood dysregulation and circadian disruption in this patient population.

**Methods:**

A non-systematic review of the literature on the melanopsin-mediated pupillary light reflex in patients with bipolar disorder, through a targeted search of databases.

**Results:**

The reviewed studies demonstrate consistent alterations in melanopsin-mediated pupillary responses in individuals with BD. Several investigations report reduced PLR amplitude and delayed latency in BD patients, with variability depending on mood state. Manic and depressive episodes appear to be associated with distinct patterns of dysregulation, suggesting that melanopsin signaling may be differentially affected by the phase of illness. One study also shows a variation in ipRGCs responses in remitted BD patients, suggesting an alteration of this system throughout the disease. (Madsen *et al*. Int J Bipolar Disord 2021;9,7) These findings align with circadian disruptions commonly observed in BD, further supporting a mechanistic link between ipRGC dysfunction and mood regulation.

**Conclusions:**

Melanopsin-mediated pupillary responses have emerged as a promising avenue of investigation for identifying biomarkers of bipolar disorder, particularly concerning circadian rhythm and mood dysregulation. Despite encouraging preliminary findings, the current literature is limited by methodological heterogeneity and small sample sizes. Future research should standardize assessment protocols and investigate how ipRGC dysfunction contributes to BD pathophysiology, potentially leading to novel treatments targeting circadian regulation and improving clinical outcomes.

**Disclosure of Interest:**

None Declared

